# Autologous Graft versus Host Disease: An Emerging Complication in Patients with Multiple Myeloma

**DOI:** 10.1155/2014/891427

**Published:** 2014-05-04

**Authors:** Anu Batra, Michele Cottler-Fox, Terry Harville, Bobbie S. Rhodes-Clark, Issam Makhoul, Mayumi Nakagawa

**Affiliations:** ^1^Division of Hematology and Oncology, Department of Medicine, University of Arkansas for Medical Sciences, 4301 West Markham Street, Slot 508, Little Rock, AR 72205, USA; ^2^Department of Pathology, University of Arkansas for Medical Sciences, 4301 West Markham Street, Slot 823, Little Rock, AR 72205, USA; ^3^Department of Pathology, University of Arkansas for Medical Sciences, 4301 West Markham Street, Slot 502, Little Rock, AR 72205, USA

## Abstract

Autologous graft versus host disease (autoGVHD) is a rare transplant complication with significant morbidity and mortality. It has been hypothesized that patients with multiple myeloma might be predisposed to autoGVHD through dysregulation of the immune response resulting from either their disease, the immunomodulatory agents (IMiDs) used to treat it, or transplant conditioning regimen. Hematopoietic progenitor cell (HPC) products were available from 8 multiple myeloma patients with biopsy-proven autoGVHD, 16 matched multiple myeloma patients who did not develop autoGVHD, and 7 healthy research donors. The data on number of transplants prior to developing autoGVHD, mobilization regimens, exposure to proteasome inhibitors, use of IMiDs, and class I human leukocyte antigen types (HLA A and B) were collected. The HPC products were analyzed by flow cytometry for expression of CD3, CD4, CD8, CD25, CD56, and FoxP3. CD3^+^ cell number was significantly lower in autoGVHD patients compared to unaffected controls (*P* = 0.047). On subset analysis of CD3^+^ cells, CD8^+^ cells (but not CD4^+^ cells) were found to be significantly lower in patients with autoGVHD (*P* = 0.038). HLA-B55 expression was significantly associated with development of autoGVHD (*P* = 0.032). Lower percentages of CD3^+^ and CD8^+^ T-cells and HLA-B55 expression may be predisposing factors for developing autoGVHD in myeloma.

## 1. Introduction

Graft versus host disease (GVHD) is a common and serious complication of allogeneic stem cell transplantation. However, it is rarely seen in recipients of autologous cells, and would likely have a very different etiology as no alloantigens are introduced to the hosts. Recently, clusters of multiple myeloma (MM) patients who developed autologous graft versus host disease (autoGVHD) after receiving autologous hematopoietic progenitor cell (HPC) transplantation have been reported [1–3]. Patients with MM maybe more susceptible to developing autoGVHD as 5 cases were reported among 223 MM patients, but none in 136 patients who underwent autologous HPC transplantation for Hodgkin disease, acute myeloid leukemia, and non-Hodgkin lymphoma developed the complication [1]. Drobyski et al., therefore, proposed that some new treatment regimens may render autologous HPC transplantation recipients susceptible to autoGVHD, reporting significantly increased cases of autoGVHD in MM patients who received a second transplant compared to those who received only a single transplant [1]. Lazarus et al. showed significantly increased expression of three markers on CD34^+^ HPC of myeloma patients who developed autoGVHD in comparison to those who did not. Such changes may have been in response to myeloma treatment regimens and further suggested possible alterations of T-cells or T-cell subsets that may have led to the development of autoGVHD [2]. Indeed, there are reports that the immunomodulatory agents (IMiDs) lenalidomide and pomalidomide inhibit proliferation and function of regulatory T-cells (Tregs), which may then be incapable of regulating the immune response in those receiving autologous stem cell transplants [4]. Further, the proteasome inhibitor bortezomib induces its antimyeloma effect through the induction of apoptosis [5, 6], which can lead to increased Th17 cells, which are associated with antifungal response and are also implicated in the pathogenesis of inflammatory autoimmune diseases [7].

A cluster of autoGVHD cases in MM patients has been seen at our institution in recent years. We wished to test the hypothesis that patients with MM may be predisposed to autoGVHD through dysregulation of immune response resulting from their disease and/or their treatments using IMiDs and/or proteasome inhibitors. We, therefore, compared these factors as well as immune cell composition of HPC products and HLA Class I types of MM patients who developed and did not develop autoGVHD.

## 2. Materials and Methods

### 2.1. Patients

We identified,based on the dates of hematopoietic progenitor cell (HPC) collection (within 15 days), eight multiple myeloma patients who developed autoGVHD after transplant and 16 matched MM patient controls who did not develop autoGVHD. All patients were either enrolled on IRB-approved research protocols or had signed an IRB-approved consent for data collection. All 8 patients with autoGVHD presented with skin rash, 6 patients had diarrhea, and 3 patients also had elevated liver enzymes. All patients had biopsy results indicating or suggestive of GVHD (5 had skin biopsy, 2 had skin and gastrointestinal biopsies, and 1 had a colon biopsy). The mean duration between the last autologous HPC transplant and the diagnosis (i.e., the date of diagnostic biopsy) of autoGVHD was 29.1 ± 10.3 days (range from 14 to 45 days). All were treated with high dose corticosteroids. Six patients who did not respond well to corticosteroids were also treated with extracorporeal photopheresis. MM patients who developed autoGVHD (*n* = 8) were well matched with the controls who did not develop autoGVHD (*n* = 16) in terms of age and racial distribution (Table 1). In the autoGVHD group, 62.5% were males, and 37.5% were males in the non-autoGVHD group. Mean age for the autoGVHD group was 60 ± 5.3 years, and 62.1 ± 7.8 years in the non-autoGVHD group. Information on the following variables was collected: number of autologous HPC transplants (prior to developing autoGVHD for the disease group, and through April 2013 for nondisease group), mobilization regimens, exposure to bortezomib, and IMiDs (thalidomide, lenalidomide, and pomalidomide).

HPC products from 7 healthy research donors (Key Biologics, LLC, Memphis, Tennessee) were also examined. The healthy HPC donors were all males and younger than the two patient groups (37 ± 7.1 years old, *P* < 0.0001).

### 2.2. Flow Cytometry

Aliquots from the HPC products were thawed and counted using a hemocytometer. Thawed cells were stained with relevant isotype controls and the following combinations of monoclonal antibodies: (1) fluorescein isothiocyanate (FITC-) labeled anti-human CD4 (clone RPA-T4), PerCP-Cy5.5-labeled anti-human CD25 (clone BC96), and allophycocyanin (APC-) labeled anti-human Foxp3 (clone PCH101) and (2) FITC-labeled anti-human CD3 (OKT3), phycoerythrin-labeled anti-human CD8 (clone OKT8), and APC-labeled anti-human CD56 (NCAM, clone MEM 188). All of the antibodies were purchased from eBioscience (San Diego, CA). Cells were first stained with antibodies for surface markers CD4, CD25, CD3, CD8, and CD56. Intracellular Foxp3 staining was performed using the Foxp3 staining kit according to the manufacturer's instructions (eBioscience). Flow cytometric analysis was performed with FACS Fortessa using FACS Diva software (Becton Dickinson Biosciences, San Jose, CA) at the University of Arkansas for Medical Sciences Microbiology and Immunology Flow Cytometry Core Laboratory. Lymphocytes were gated and 10,000 events were analyzed per sample. NK cells were defined as CD3^−^CD56^+^. Tregs were defined as CD4^+^CD25^+^Foxp3^+^ and the frequency was expressed as a percentage of CD4^+^CD25^+^Foxp3^+^/CD4^+^ [8].

### 2.3. HLA Typing

HLA typing (class I A and B) was performed at the University of Arkansas for Medical Sciences HLA laboratory using peripheral blood leukocytes or small aliquots of HPC products using DNA-based methodologies including a reverse sequence-specific oligonucleotide probe method. Commercial kits were purchased from One Lambda, Inc. (Canoga, Park, CA), and the assays were performed as recommended by the manufacturer. Briefly, specific biotinylated primers were used to amplify exons 2 and 3 for each class I locus. The resultant amplified DNA was denatured and hybridized to oligonucleotide probes attached to unique microsphere beads for identification of the HLA types. The biotinylated, amplified DNA product was detected using R-Phycoerythrin-conjugated Streptavidin (SAPE). The DNA hybridized to specific oligonucleotides attached to specific microspheres and detected by SAPE were analyzed on the LABScan 100 flow analyzer from Luminex technology (Austin, TX). The extent of SAPE bound and the pattern of probe binding were used to assign the specificity of each HLA type expressed [9], and an HLA Fusion software provided by One Lambda, Inc. was used for analysis. The HLA gene frequencies of the population in the United States were calculated using information from the Organ Procurement and Transplantation Network [10].

### 2.4. Statistical Analyses

One-way ANOVA was used to compare ages among those in the MM autoGVHD, MM non-autoGVHD, and healthy control groups. Mann-Whitney test was used to compare number of autologous HPC transplants between the autoGVHD and non-autoGVHD groups. Unpaired *t*-test was used to compare the percentages of CD3, CD4, CD8, NK, and Tregs, and Fisher's exact test was used to compare HLA gene frequencies between the two groups. A *P* value < 0.05 was considered significant.

## 3. Results

**Figure 1:**
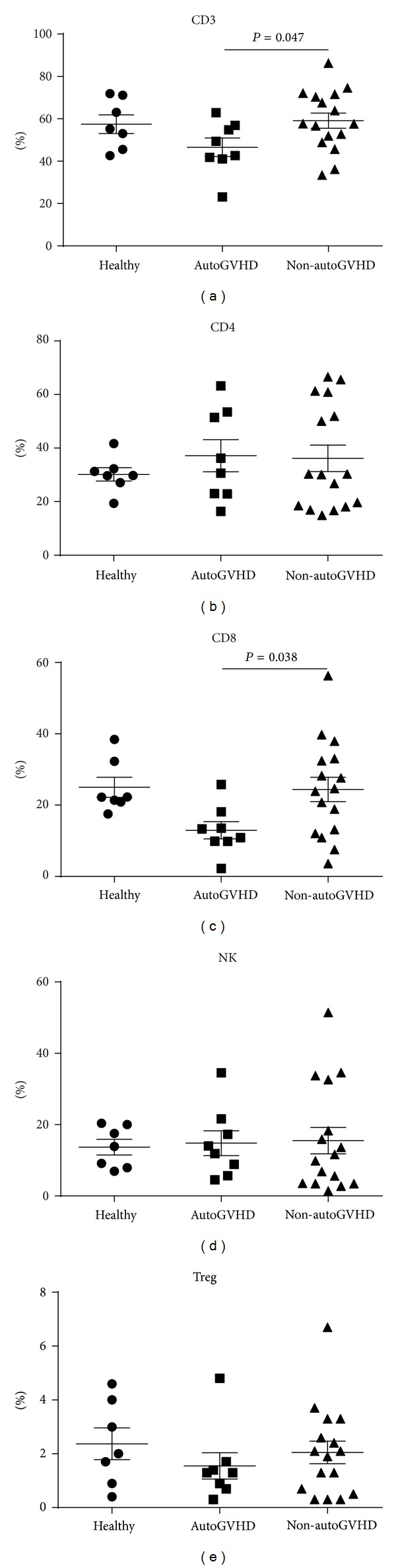
Immune parameters of HPC products among the healthy control group, the group of MM patients who developed autoGVHD, and the group of MM patients who did not develop autoGVHD. Mean percentages of lymphocytes are shown for CD3^+^, CD4^+^, CD8^+^, and NK (CD3^−^ CD56^+^). Mean percentages of CD4-positive T-cells that are FoxP3^+^/ CD25^+^ are shown for Tregs. The bars represent standard error of means.

The myeloma patients who developed autoGVHD and those who did not were similar in mobilization regimens received (Table 1), exposure to bortezomib (all patients in both groups), and treatment with IMiDs (8 of 8 patients in the autoGVHD group and 14 of 16 patients in the non-autoGVHD group received thalidomide; 2 of 8 patients in autoGVHD group and 7 of 16 patients in the non-autoGVHD group received lenalidomide; only one patient in the non-autoGVHD group received pomalidomide). The autoGVHD group received a larger number of transplants (2.1 ± 0.8, SD) compared to the non-autoGVHD group (1.6 ± 0.7, SD), but the difference was not statistically significant. The clinical course of each patient is summarized in terms of therapies received prior to stem cell collection from which an aliquot was used for analysis (Figure 1), months between the time of MM diagnosis and stem cell collection, days between transplant and the time of autoGVHD diagnosis if applicable, and the latest disease status (Table 2).

The percentages of CD3^+^, CD4^+^, CD8^+^, NK cells, and Tregs in HPC products were compared among the healthy controls, autoGVHD group, and non-autoGVHD group (Figure 1). The CD3^+^ and CD8^+^ levels were significantly lower in the autoGVHD group compared with the non-autoGVHD group (*P* = 0.047 and 0.038, resp.). The CD4^+^ and NK levels did not differ. Treg levels were lower in the autoGVHD group (1.6% ± 0.5%, SEM) compared with the non-autoGVHD group (2.1% ± 0.4%, SEM), but the difference was not statistically significant.

**Figure 2:**
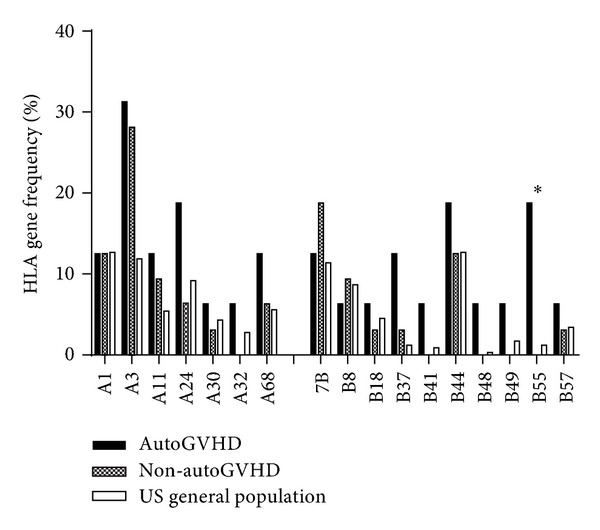
HLA class I gene frequencies among MM patients who developed and those who did not develop autoGVHD and the general population. ∗ indicates a statistically significant difference for B55 expression between MM patients who developed and did not develop autoGVHD (*P* = 0.032).

The comparisons of HLA gene frequencies among MM patients who developed autoGVHD, those who did not develop autoGVHD, and the US general population (Figure 2) showed increased frequencies of B37 and B55 in the MM autoGVHD group compared to the other two groups. The B55 gene frequency of MM patients who developed autoGVHD (3 of 16) and that of MM patients who did not develop autoGVHD (none of 32) were significantly different (*P* = 0.032). The same comparison for B37 was not significant.

## 4. Discussion

Autologous GVHD is an emerging, rare but serious complication seen in patients with MM. As reported by Drobyski and colleagues [1], we have seen this complication only in patients with MM although it should be noted that the number of patients with other diagnoses in whom we perform autologous stem cell transplantation (e.g., non-Hodgkin lymphoma) is somewhat smaller. The nature and mechanisms of autoGVHD are still under investigation. It would not be surprising, if it is an autoimmune phenomenon, as autoimmune complications are seen in this setting. Autoimmune thyroiditis with detectable anti-thyroid peroxidase antibody after autologous transplantation has been reported in an MM patient [11], as have exacerbations of underlying autoimmune diseases [12]. Dhodapkar et al. [7] have reported a higher percentage of Th17 cells in the bone marrow of patients with MM compared to patients with monoclonal gammopathy of undetermined significance. This may predispose MM patients to develop autoGVHD, as these Th17 cells are implicated in the pathogenesis of inflammatory autoimmune diseases [7]. MM can be considered as a Th2 disorder because of the increased B lymphocyte activity. Therefore, it may be natural for the immune system to counteract this increase in Th2 activity by decreasing the Th2 activity and increasing theTh1/17 activity. Again, the increase in Th17 may predispose to the development of autoimmunity.

Alternatively, newer treatments for MM may have inadvertently promoted the development of autoGVHD in some patients. All the cases of autoGVHD in MM patients at the Medical College of Wisconsin have been occurring since 2004 [1], and the first case at our institution was diagnosed around 2009. The use of bortezomib in Phase I trial was first published in 2002 [13], and it has been shown to induce apoptosis [5, 6]. This may lead to the development of autoimmunity since uptake of apoptotic myeloma tumor cells by dendritic cells has been shown to lead to enhanced induction of Th17 cells [7]. Lenalidomide and pomalidomide have been reported to inhibit the proliferation and function of Tregs [4], and the combination of effects by proteasome inhibitors and IMiDs may predispose some patients to develop autoGVHD. However, no difference between the group of MM patients who developed or did not develop autoGVHD was found in the use of bortezomib, lenalidomide, pomalidomide, and thalidomide in our study. Therefore, if these drugs are involved in the pathogenesis of autoGVHD, additional factors are likely to be involved. Unlike what was reported by Drobyski and colleagues [1], there was no difference in the number of transplants received in the autoGVHD group compared with the non-autoGVHD group. It is possible that autologous transplant, regardless of how many, may be an additional factor leading to autoGVHD as autoimmune thrombocytopenia and autoimmune hemolytic anemia have also been reported in patients who received autologous stem cell transplantation for indications other than MM [14, 15].

We found significant differences in the immune parameters, specifically CD3^+^ and CD8^+^ T-cells in HPC products between MM patients who did and those who did not develop autoGVHD (*P* = 0.047 and 0.038, resp.) (Figure 1). No difference was found between the two groups in terms of percentages of CD4^+^ T-cells, NK cells, and Tregs. Since the percentage of Tregs in the affected MM patients was lower compared to in the unaffected MM patients, it is possible that examining a larger number of patients may reveal the role of Tregs in the pathogenesis of autoGVHD in myeloma. Following mobilization, the percentage of CD8^+^ T-cells appears to be decreased in susceptible individuals, which may be a factor predisposing them to develop autoimmunity, that is, autoGVHD. This may be due to a compensatory mechanism by autoreactive immune cells in response to mobilization regimens, which have resulted in lower CD8^+^ T-cell levels. Lazarus and colleagues also examined immune parameters of CD34^+^ and found significantly increased expression of GATA-2 (an HPC transcription factor expressed that promotes proliferation [16]), CD130 (interleukin-6 receptor), and CXCR4 (a chemokine receptor specific for stromal-derived-factor-1) on CD34^+^ HPC of 9 autoGVHD patients, compared with 42 non-autoGVHD patients using enzymatic amplification staining-enhanced flow cytometry [2]. The significance of such increased expressions in HPC is not clear, although the authors allude to their effects on T-cells and T-cell subsets [2]. An interesting area of further investigation would be to look into whether the increases in the 3 markers on CD34^+^ HPC are associated with the decreased CD3^+^ and CD8^+^ T-cells in the same patients. Drobyski and colleagues examined the percentages of CD4^+^, CD8^+^, and Tregs between 3 MM patients who developed autoGVHD and 12 patients who did not develop autoGVHD and found no significant differences [1].

HLA associations have been reported for many autoimmune diseases. A well-known example is the association between the presence of HLA-B27 and ankylosing spondylitis, a chronic arthritis of the spine and the sacroiliac joints in the pelvis. At least a part of pathogenesis in this disorder appears to be mediated by presentation of autologous peptides by the B27 molecule [17–20]. In this study, we found a significant association between HLA-B55 expression and the development of autoGVHD in MM patients (*P* = 0.032) raising a possibility that the presentation of autoantigen by the B55 molecule may be a part of pathogenesis of autoGVHD. HLA-B55 is a relatively less common HLA type, more generally found in the Asian population with the average frequency of 3.67% [21]. The fact that none of the autoGVHD MM patients were Asian strengthens the possibility that B55 may somehow contribute to the pathogenesis of autoGVHD.

In conclusion, we found decreased percentages of CD3^+^ and CD8^+^ T-cells in HPC products and host expression of HLA-B55 to be correlated with the development of autoGVHD in MM patients and therefore suggest that these parameters may be predisposing factors for developing this clinical entity.

## Figures and Tables

**Table 1:** MM patient characteristics.

Characteristic	AutoGVHD Number (*n* = 8)	Non-autoGVHD Number (*n* = 16)
Age, years		
Mean ± SD	60 ± 5.3	62.1 ± 7.8
Range	51–66	42–71
Sex		
% male	62.5	37.5
Race, number		
Caucasian	7	15
African American	0	1
Hispanic	1	0
Mobilization, number		
G	0	2
DTPACE + G	1	1
BDTPACE + G	3	5
MBDTPACE + G	3	5
DTPACE + pegG/DA	1	0
BDTPACE + PL + G	0	3
Autologous HPC transplant, number		
Mean ± SD	2.1 ± 0.8	1.6 ± 0.7

A: adriamycin; B: bortezomib; C: cyclophosphamide; D: dexamethasone; DA: darbepoetin alfa; E: etoposide; G: granulocyte-colony stimulating factor; M: melphalan; P: cisplatin; PL: plerixafor; SD: standard deviation; T: thalidomide.

**Table 2:** Clinical summaries of MM patients who developed autoGVHD (1–8) and who did not (9–24).

Patient	Age	Sex	Race	Prior therapy	Time from MM diagnosis to collection (months)	Mobilization	Time from txp to autoGVHD diagnosis (days)	Disease course
1	66	M	CA	B, A, and D	2	BDTPACE + G	33	autoGVHD between ttxp, WR, died of sepsis 1 month after 2nd txp

2	62	F	CA	None	5	DTPACE + G	35	autoGVHD after ttxp and WR, died of pneumonia 6 months later

3	56	M	H	T, D, XRT, and B	9	DTPACE + pegG/DA	28	txp, WR, and autoGVHD after 2nd txp 5 years later, near remission, died in home state 3 months later

4	63	F	CA	None	3	MBDTPACE + G	32	autoGVHD after ttxp and CR

5	66	M	CA	B, A, and D	12	MBDTPACE + G	45	autoGVHD after ttxp and CR, died of PCP pneumonia 5 months later

6	60	M	CA	B, A, D, XRT, and L	4	BDTPACE + G	15	autoGVHD after 4 of 5 txp and WR, died in home state the following year

7	51	F	CA	None	2	BDTPACE + G	14	autoGVHD after ttxp and WR, died in home state 11 months later

8	56	M	CA	B, D	1	MBDTPACE + G	31	autoGVHD after ttxp and CR

9	61	F	CA	Z	40	MBDTPACE + G	NA	txp and WR, died of pneumonia 1 month later

10	56	F	CA	D, T, B, M, P, A, C, E, L, CAR, CT, M, HBO, and txp	42	BDTPACE + PL + G	NA	WR, died of progressive disease

11	64	F	CA	D, T, M, 2 txp, L, B, A, C, E, G, PL, P, and Z	62	BDTPACE + PL + G	NA	txp and WR

12	63	M	CA	None	2	MBDTPACE + G	NA	ttxp and WR, died of multiorgan failure 25 months later

13	66	F	CA	B and A	27	BDTPACE + G	NA	txp and WR, died of RSV pneumonia the same month

14	54	F	CA	B, C, D, G, M, and 2 txp	13	BDTPACE + PL + G	NA	CR and relapse, died of sepsis

15	68	M	CA	None	1	MBDTPACE + G	NA	ttxp and CR

16	59	M	CA	B, A, D, M, 2 txp, T, and L	108	MBDTPACE + G	NA	txp and WR, died of sepsis 45 months later

17	42	F	CA	B and D	2	G	NA	ttxp, status not known

18	66	F	CA	XRT	2	BDTPACE + G	NA	ttxp and WR, died of pneumonia 1 month later

19	52	F	AA	D	2	MBDTPACE + G	NA	ttxp and CR

20	66	M	CA	None	2	BDTPACE + G	NA	ttxp and CR

21	66	F	CA	B, A, D, C, E, P, PL, T, and PA	28	BDTPACE + G	NA	txp and WR

22	71	M	CA	None	2	BDTPACE + G	NA	txp and CR

23	69	F	CA	None	1	DTPACE + G	NA	ttxp and WR, died of pneumonia 1 month later

24	70	M	CA	B and D	12	G	NA	WR

A: adriamycin; AA: African American; B: bortezomib; C: cyclophosphamide; CA: Caucasian; CAR: carmustine; CR: complete remission; CT: cytarabine; D: dexamethasone; DA: darbepoetin alfa; E: etoposide; G: granulocyte-colony stimulating factor; H: Hispanic; HBO: hyperbaric oxygen; L: lenalidomide; M: melphalan; NA: not applicable; P: cisplatin; PA: pamidronate; PL: plerixafor; T: thalidomide; txp: single autologous stem cell transplant; ttxp: tandem autologous stem cell transplants (within 12 months); WR: without remission; XRT: local radiation therapy; Z: zoledronic acid.

## References

[1] Drobyski WR, Hari P, Keever-Taylor C, Komorowski R, Grossman W (2009). Severe autologous GVHD after hematopoietic progenitor cell transplantation for multiple myeloma. *Bone Marrow Transplantation*.

[2] Lazarus HM, Sommers SR, Arfons LM (2011). Spontaneous autologous graft-versus-host disease in plasma cell myeloma autograft recipients: flow cytometric analysis of hematopoietic progenitor cell grafts. *Biology of Blood and Marrow Transplantation*.

[3] Fidler C, Klumpp T, Mangan K (2012). Spontaneous graft versus host disease occurring in a patient with multiple myeloma after autologous stem cell transplant. *American Journal of Hematology*.

[4] Galustian C, Meyer B, Labarthe M-C (2009). The anti-cancer agents lenalidomide and pomalidomide inhibit the proliferation and function of T regulatory cells. *Cancer Immunology, Immunotherapy*.

[5] Ling Y-H, Liebes L, Ng B (2002). PS-341, a novel proteasome inhibitor, induces Bcl-2 phosphorylation and cleavage in association with G2-M phase arrest and apoptosis. *Molecular Cancer Therapeutics*.

[6] Hideshima T, Richardson P, Chauhan D (2001). The proteasome inhibitor PS-341 inhibits growth, induces apoptosis, and overcomes drug resistance in human multiple myeloma cells. *Cancer Research*.

[7] Dhodapkar KM, Barbuto S, Matthews P (2008). Dendritic cells mediate the induction of polyfunctional human IL17-producing cells (Th17-1 cells) enriched in the bone marrow of patients with myeloma. *Blood*.

[8] Kim KH, Greenfield WW, Cannon MJ, Coleman HN, Spencer HJ, Nakagawa M (2012). CD4^+^ T-cell response against human papillomavirus type 16 E6 protein is associated with a favorable clinical trend. *Cancer Immunology, Immunotherapy*.

[9] Harville T, Areman EM, Loper K (2009). HLA typing for cellular product characterization and identity testing. *Cellular Therapy: Principles, Methods, and Regulations*.

[10] Organ Procurement and Transplantation Network

[11] Ishikawa F, Shigematsu H, Gondo H, Okamura T, Niho Y (1998). Autoreactive antibodies following autologous peripheral blood stem cell transplantation. *Bone Marrow Transplantation*.

[12] Isshiki I, Okamoto S, Kakimoto T (2006). Recurrence of autoimmune disease after autologous peripheral blood stem cell transplantation for multiple myeloma. *International Journal of Hematology*.

[13] Orlowski RZ, Stinchcombe TE, Mitchell BS (2002). Phase I trial of the proteasome inhibitor PS-341 in patients with refractory hematologic malignancies. *Journal of Clinical Oncology*.

[14] Ahmad I, Haider K, Kanthan R (2004). Autoimmune thrombocytopenia following tandem autologous peripheral blood stem cell transplantation for refractory germ cell tumor. *Bone Marrow Transplantation*.

[15] Lambertenghi-Deliliers G, Annaloro C, della Volpe A, Oriani A, Pozzoli E, Soligo D (1997). Multiple autoimmune events after autologous bone marrow transplantation. *Bone Marrow Transplantation*.

[16] Briegel K, Lim K-C, Plank C, Beug H, Engel JD, Zenke M (1993). Ectopic expression of a conditional GATA-2/estrogen receptor chimera arrests erythroid differentiation in a hormone-dependent manner. *Genes & Development*.

[17] Benjamin R, Parham P (1990). Guilt by association: HLA-B27 and ankylosing spondylitis. *Immunology Today*.

[18] Colbert RA (2000). HLA-B27 misfolding: a solution to the spondyloarthropathy conundrum?. *Molecular Medicine Today*.

[19] Colbert RA, Tran TM, Layh-Schmitt G (2014). HLA-B27 misfolding and ankylosing spondylitis. *Molecular Immunology*.

[20] Edwards JCW, Bowness P, Archer JR (2000). Jekyll and Hyde: the transformation of HLA-B27. *Immunology Today*.

[21] Marsh S, Parham P, Barber L (2000). *The HLA FactsBook*.

